# Comparing skin swabs, buccal swabs, and toe clips for amphibian genetic sampling, a case study with a small anuran (*Acris blanchardi*)

**DOI:** 10.1093/biomethods/bpae030

**Published:** 2024-05-16

**Authors:** Travis A Rainey, Emily E Tryc, Kirsten E Nicholson

**Affiliations:** Department of Biology, Central Michigan University, Mount Pleasant, MI 48859, United States; Department of Biology, Central Michigan University, Mount Pleasant, MI 48859, United States; Department of Biology, Central Michigan University, Mount Pleasant, MI 48859, United States

**Keywords:** Blanchard’s Cricket Frog, DNA extraction, genotyping, non-invasive sampling, non-lethal sampling

## Abstract

Multiple methods for collecting genetic samples from amphibians exist, each with their own implications for study design, animal welfare, and costs. Toe clipping is one common method, but there is ongoing debate regarding its potential detriment. Less invasive methods should be implemented, if efficacious, as amphibians are a particularly vulnerable vertebrate group. Skin and buccal swabbing are less invasive methods for genetic sampling, but the potential for contamination and a lower yield of DNA may exist. To compare these methods, we gathered skin swabs, buccal swabs, and toe clips from the same individuals of a relatively small anuran species, Blanchard’s Cricket Frog (*Acris blanchardi*). We then compared DNA yield, DNA purity, amplification success rate, and genotypic data quality among sample types. We found toe clips and buccal swabs generated similar DNA yield and purity, with skin swabs yielding significantly less DNA of significantly lower purity than the other sample types. Amplification success rate was significantly higher using toe clips compared to the other sample types, though buccal swab samples amplified more readily than skin swabs. Genotypic data from toe clips and buccal swabs did not differ significantly in quality, but skin swab data quality was significantly lowest among sample types. Thus, skin swabbing could produce erroneous data in some situations, but buccal swabbing is likely an effective substitute to toe clipping, even for small species. Our results can help future researchers select which genetic sampling method might best suit their research needs.

## Introduction

Refining animal research methodology to minimize pain and maximize the welfare of study organisms has long been avowed [1]. In genomic fields, technological advancements have allowed nonlethal forms of DNA sampling to become more prevalent over recent decades [2–4]. Nonlethal genetic sampling of herpetofauna may involve the collection of toe or tail clips (considered herein as tissue sampling; outlined in Heyer *et al*. [5]), while other methods use swabs (considered herein distinct from tissue sampling), such as buccal swabbing [6, 7], cloacal swabbing [8, 9], and skin/dermal swabbing [10, 11]. Swabbing methods do not fit the common definition of “non-invasive” sampling because they still require handling animals [12, 13], and due to their size, phenology, and cryptic nature, most amphibians are not well suited for non-invasive sampling [14, 15]. However, while swabbing methods may not be non-invasive, they are less invasive than clipping methods [6, 14], and nonlethal and less invasive sampling methods are preferred over lethal or invasive methods [16].

A review of recent studies employing DNA sampling methods suggested toe and tail clipping are the most common nonlethal methods for genetic sampling of amphibians, respectively, accounting for 32% and 18% of studies, whereas buccal swabbing and skin swabbing were implemented just 12% and 2% of the time, respectively, with the remaining 34% of studies employing lethal sampling methods [17]. Despite its commonality, the ethics of toe clipping remains contested in the literature [16, 18], given that it may reduce survival [19, 20] and hinder motility and endurance [21] for some species, although some of the impacts may be due to handling alone [22]. Conversely, most work suggests little or no negative effect of toe clipping amphibians [23], including recent assessments [24, 25], and it has been suggested that stress associated with swabbing could cause greater disturbance than tissue collection in certain circumstances [26, 27].

Like toe clips, swabbing methods have a mix of benefits and limitations reported in the literature. Why these swabbing techniques, which have been available for decades [14, 28–30], remain less common than more invasive sampling could be due to concerns regarding the potential for contamination, additional costs of specialized extraction kits, and their collection of low DNA quantity and quality resulting in genotyping errors [31–36]. For example, it has been argued skin swabbing is easier and less harmful to animals than buccal or tissue sampling [33, 36], but this method might be difficult for species with drier skin and more subject to contamination from microbiota or conspecifics [32, 33, 37]. However, skin swabbing has been seemingly successfully used in many recent population genetics [11, 38], phylogeography [39], and other studies [40, 41].

Buccal swabbing has been shown to yield DNA with quality comparable to that yielded from tissues [14, 34, 42] and quantities suitable for next-generation sequencing [27], but it has been suggested that this method could be stressful or injurious for small-mouthed species or species resistant to opening their mouths [15, 33, 37]. Furthermore, this method can yield less DNA than tissues [34], and a reptile study found the type of swab and DNA extraction method used can affect buccal swabbing success [35]. Despite these concerns, buccal swabbing has recently been used for collecting phylogeographic [43, 44], and population and conservation genetic data [45–47].

Amplification success rate and DNA yield from swabbing methods for amphibians have been evaluated [27, 30, 48, 49], but because these studies did not take samples from the same individuals, direct comparisons of downstream genotypic data quality between sampling types could not be made. Several studies have directly compared various sample types taken from the same individuals [14, 32–34, 37, 42, 50, 51], but to our knowledge, no study to date has analytically compared amphibian tissue samples, buccal swabs, and skin swabs sourced from the same sample of individuals. Additionally, there appears to be a lack of consensus on the efficacy of swabbing methods across these previous studies.

Herein, we assess the viability of toe clips, buccal swabs, and skin swabs, taken in a field setting from the same individuals, for genetic sampling. We quantify and compare DNA yield and purity, amplification success rate, and the rate of downstream genotyping error of these sampling methods. This study also helps fill in additional knowledge gaps related to skin and buccal swabbing of small anurans, as we sampled Blanchard’s Cricket Frog (*Acris blanchardi*), a species with mean snout-vent length near 24 mm for males and 26 mm for females [52]. The only other study testing the efficacy of skin swabbing with such a small amphibian species found the method to be ineffective, which the author attributed to the focal species being a dry-skinned, terrestrial anuran [33]. By skin swabbing the Blanchard’s Cricket Frog, which is non-climbing and considered the most aquatic Hylid in North America [52, 53], we can also speak to the viability of skin swabbing a small amphibian with moist skin. Furthermore, while previous studies have obtained genetic data from buccal swabbing anurans of size comparable to Blanchard’s Cricket Frogs [7, 42, 43, 45] or even smaller [34, 46], almost no evidence is reported addressing the notion posed by Ringler [33] that this method could be harmful for small species. Only one paper we found mentions buccal swabbing’s impact on small amphibians; Mudke *et al*. [46] confirm that four Kottigehar Dancing Frogs (*Micrixalus kottigeharensis*) showed no signs of discomfort after buccal swabbing, although frogs were immediately released after sampling. Thus, along with the main objective of comparing genetic sampling sourced from skin swabs, buccal swabs, and tissues, we can also help fill in the knowledge gap regarding the impact of buccal swabbing small amphibians.

## Materials and methods

### Sampling

We collected toe clips, buccal swabs, and skin swabs from adult Blanchard’s Cricket Frogs during opportunistic encounter surveys conducted for a separate study [54], across the southern Lower Peninsula of Michigan, USA. Sampling took place between 16 May and 13 July 2022, during the species breeding season in Michigan, when breeding congregations and male calling facilitated locating individuals. We clipped right posterior toes in the fourth position with sterile, stainless-steel scissors above the attachment of webbing (roughly one or two phalanges, proximally, from the tip). We applied a combined antiseptic (0.13% w/w Benzalkonium Cl), analgesic (4% w/w Lidocaine HCl) spray with a sterile tissue to the location of the clipped toe. We firmly passed skin swabs (sterile, cotton-tipped; Concordance Healthcare Solutions HCS1002, USA) five times along the dorsal and ventral sides, both ventral sections of thighs, while rolling swabs in the opposite direction of swabbing during each pass, similar to other studies employing skin swabbing [32, 37] with additional passes for small body size. We gently rotated buccal swabs (sterile, flocked, nylon-tipped; GoBioMed GBM-9600, USA) four to five times on both sides of the mouth while passing along cheeks and tongue, similar to other studies employing buccal swabbing [27, 30]. Buccal swabs were checked for blood, and frogs were observed for 10–30 min. We placed tissues and swab tips in 95% ethanol and stored them at −80°C prior to extraction.

### DNA extraction

We used the same DNA extraction protocol [55] for all sample types and completed all extractions within 6 months of sample collection. We placed samples (toes and swab tips) in 250 µl of lysis buffer and 15 µl of Proteinase K in a microcentrifuge tube and then incubated extraction tubes at 37°C for 12–16 h. We then vortexed samples, removed swab fibers from the lysing solution, added 10 µl of 5M NaCl and 500 µl 80% isopropanol, before centrifuging at 13,300 rpm for 45 min to facilitate the precipitation of DNA pellets. We discarded supernatant and added 1000 ml of 70% ethanol, before centrifuging for another 45 min at 13,300 rpm. We discarded ethanol and allowed the resulting DNA pellets to air dry before their resuspension in 150 µl TE buffer (Tris and EDTA). We stored template at 4°C for 24 h before DNA quantification.

### DNA yield and purity

Using a NanoDrop ND-1000 Spectrophotometer (Thermo Fisher Scientific, Wilmington, Delaware, USA; “NanoDrop” hereafter), we measured DNA concentration (ng/µl; later converted to mass, or “DNA yield”) and DNA purity (the ratio of spectrophotometer absorbance at 260 nm to 280 nm; “260/280 ratio” hereafter). The 260/280 ratio measures contamination by proteins and other contaminants with an absorbance of 280 nm, and values near 1.8 are considered “pure” [56]. We took these measurements in triplicate and used the triplicate mean for analyses, as imprecision in NanoDrop measurements at low DNA concentrations has been reported [57, 58]. Between individuals, we blanked the NanoDrop with TE buffer (DNA resuspension solution), as a reference measurement.

Based on Shapiro–Wilk tests, using the dplyr R package [59], only two (DNA yield and 260/280 ratio for tissue samples) of the six datasets (three sample types by two NanoDrop variables) were normally distributed (all other *W *≤* *0.940 and *P *≤* *.034), so we used nonparametric Kruskal–Wallis tests to identify differences in mean ranked DNA concentration and purity between sampling types. To identify significant pairwise differences between sample types, we performed Dunn’s post hoc tests with a Bonferroni corrected significance level (α = 0.0167; based on the 0.05 a priori significance level used throughout all analyses herein) with the FSA package [60] in R. We performed statistics and plotting with R version 4.1.1 [61] and the ggplot2 package [62] in RStudio version 2021.09.0 + 351 [63].

### Amplification success and genotyping error

We amplified the DNA template at four microsatellite markers (Acr-3, Acr-28, Acr-2, and Acr-14) fluorescently labeled with Applied Biosystems DS-33 set (NED, 6-FAM, 6-FAM, and VIC dyes, respectively), following the program outlined by Beauclerc *et al*. [64], via polymerase chain reaction (PCR). We ran all four primers together in one multiplex reaction, using Type-It Microsatellite PCR Kits (Qiagen, Germantown, Maryland, USA), following the manual for reagent concentrations and volumes of reagents (Type-it Microsatellite PCR Handbook, October 2010). We conducted fragment analysis on an Applied Biosystems 3730xl (outsourced to North Carolina State University’s Genomics Sciences Lab), using Applied Biosystems 600 LIZ^™^ dye Size Standard version 2.0. We scored allele peaks in GeneMarker version 1.85 (SoftGenetics, LLC, State College, Pennsylvania, USA).

We considered amplification success for a sample at a given microsatellite marker binary, determined by whether PCR allowed for allele scoring, with clear homozygote or heterozygote peaks visible and near or within the expected base pair range, as per Beauclerc *et al*. [64]. We calculated the proportion of successful amplifications for each sample type. If allele peaks were not scorable based on the size standard failing to provide reference peaks, we attributed this to the failure of the Applied Biosystems 3730xl to inject PCR product into the capillary during electrokinetic injection for that sample, and we did not count these occurrences as amplification failure. For samples that experienced injection failure, we attempted one more multiplex PCR reaction. We then used pairwise Fisher’s exact tests with Bonferroni correction (adjusted α = 0.0167) to compare proportions of amplification success between sample types with the fmsb R package [65].

To quantify genotyping errors, we first calculated a baseline error rate by repeating the four-locus multiplex genotyping for a subset of 24 tissue samples, with the expectation that instances of tissue–tissue mismatch would be near zero. Using allelic data from successful swab sample amplifications, we then calculated the genotyping error rate (proportion of allelic data mismatching tissue data) for each sampling method. If tissue–tissue comparisons did not match for a given individual–locus combination, comparisons between tissues and swabbing methods were not performed for that individual–locus combination. We counted the instances of false alleles (amplification of an allele from a swab sample not observed in a corresponding tissue) and allelic dropout (failure of a swab sample to amplify one of two heterozygote alleles of a corresponding tissue sample). We conducted Bonferroni-corrected (adjusted α = 0.0167), pairwise Fisher’s exact tests to compare the proportion of genotyping error between sample types.

## Results

### Sampling, DNA yield, and purity

We collected toe clips and skin swabs from 43 individuals (39 males, 4 females), as well as buccal swabs from 39 (35 males, 4 females) of those individuals. No individuals appeared to be harmed by sampling; individuals returned to moving and calling immediately following processing. Blanchard’s Cricket Frogs readily opened their mouths by gently running a thumb down the throat, so we needed no flat prying object to induce mouth opening as suggested by previous work [14, 30]. No bleeding from buccal swabbing was observed, as has been reported with other species [14], nor did toe clipping cause observable bleeding.

**Figure 1.**
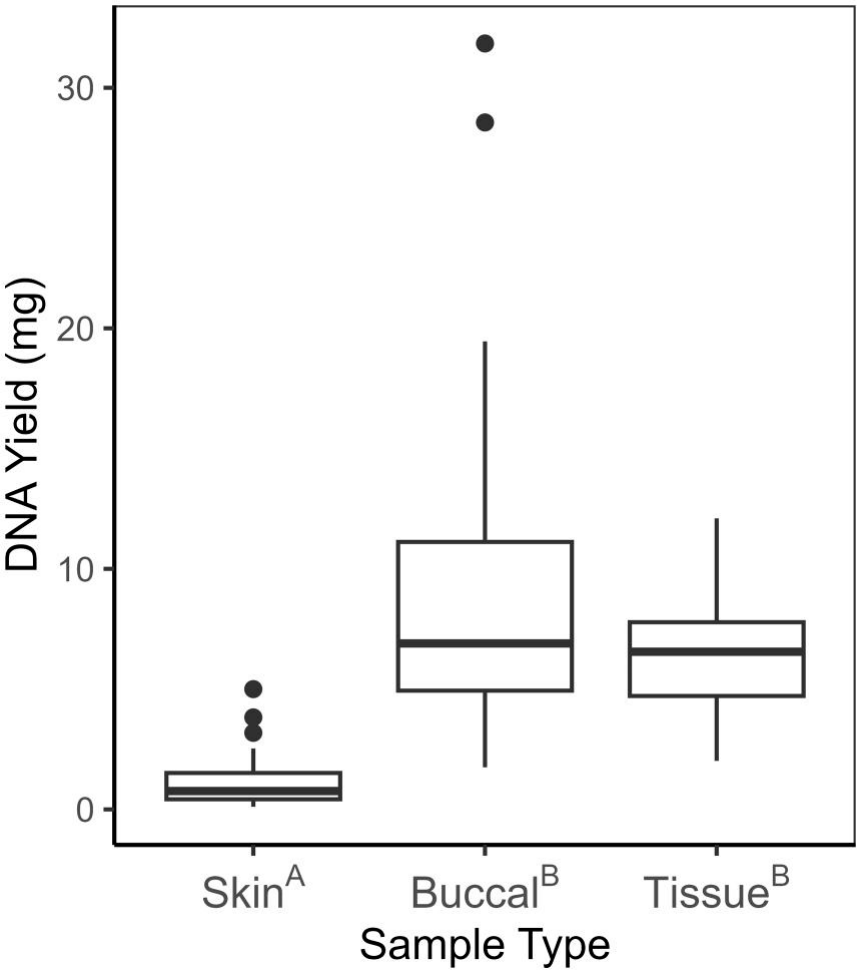
The distribution of DNA yield (mg) by sample type (skin and buccal swabs, tissue from toe clips), for Blanchard’s Cricket Frog (*A. blanchardi*) samples, based on concentration measurements by NanoDrop. Superscript letters after sample types indicate significant pairwise differences in yield between sample types, based on pairwise Dunn tests with Bonferroni correction (*P *≤* *.017). Medians symbolized by thick horizontal lines within boxes, box minimums indicate lower (Q_1_), box maximums indicate upper quartiles (Q_3_), whisker ends indicate distribution minimums and maximums, not including outliers, which are plotted individually and defined as values above Q_1_+1.5(R_IQ_) or below Q_3_(R_IQ_), where R_IQ_—interquartile range.

The DNA yielded from the three sample types differed significantly overall, based on the Kruskal–Wallis test (*H *=* *81.015, df = 2, *P *<* *.001). Skin swabs yielded significantly less DNA (median = 0.76 mg) than buccal swabs (*Z *=* *−8.124, *P *<* *.001) and toe clips (*Z *=* *−7.347, *P *<* *.001) based on Dunn’s post hoc tests (Fig. 1). Buccal swabs yielded a median of 6.55 mg of DNA across samples, which did not significantly differ (*Z *=* *0.954, *P *=* *.340) from toe clips, which yielded a median of 6.90 mg of DNA (Fig. 1).

**Figure 2.**
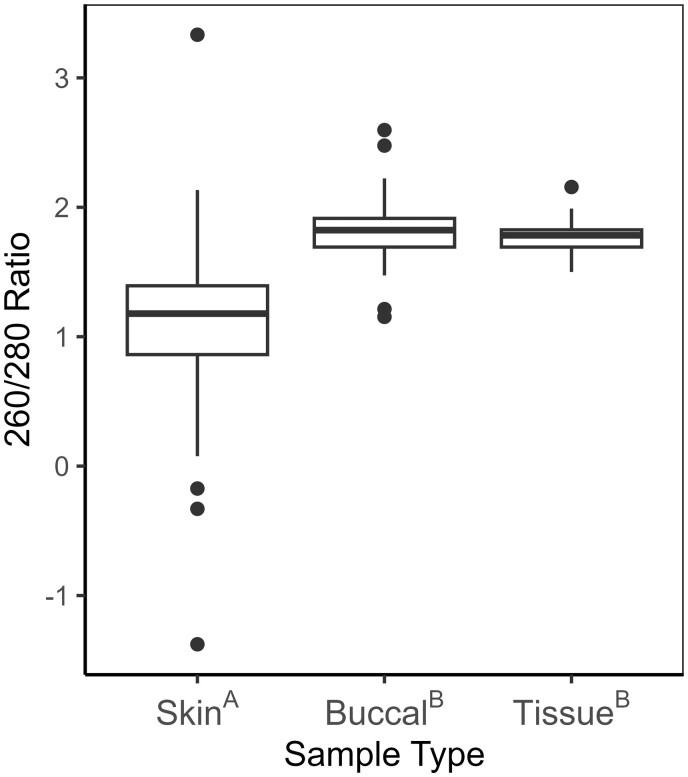
The distribution of 260/280 ratio measurements by sample type (skin and buccal swabs, tissue from toe clips), for Blanchard’s Cricket Frog (*A. blanchardi*) samples, measured by NanoDrop. Superscript letters after sample types indicate significant pairwise differences in 260/280 ratio between sample types, based on pairwise Dunn tests with Bonferroni correction (*P *≤* *.017). Medians symbolized by thick horizontal lines within boxes, box minimums indicate lower (Q_1_), box maximums indicate upper quartiles (Q_3_), whisker ends indicate distribution minimums and maximums, not including outliers, which are plotted individually and defined as values above Q_1_+1.5(R_IQ_) or below Q_3_(R_IQ_), where R_IQ_—interquartile range.

The 260/280 ratio differed significantly among sample types (*H *=* *59.206, df = 2, *P *<* *.001). Skin swab samples had a median 260/280 ratio of 1.178, which was significantly lower than buccal swabs (*Z *=* *−7.009, *P *<* *.001) and tissues (*Z *=* *−6.192, *P *<* *.001), which had median 260/280 ratios of 1.823 and 1.783, respectively (Fig. 2). The 260/280 ratios of buccal swabs and tissue samples did not significantly differ (*Z *=* *0.966, *P *=* *.334).

### Amplification success and genotyping error

**Figure 3.**
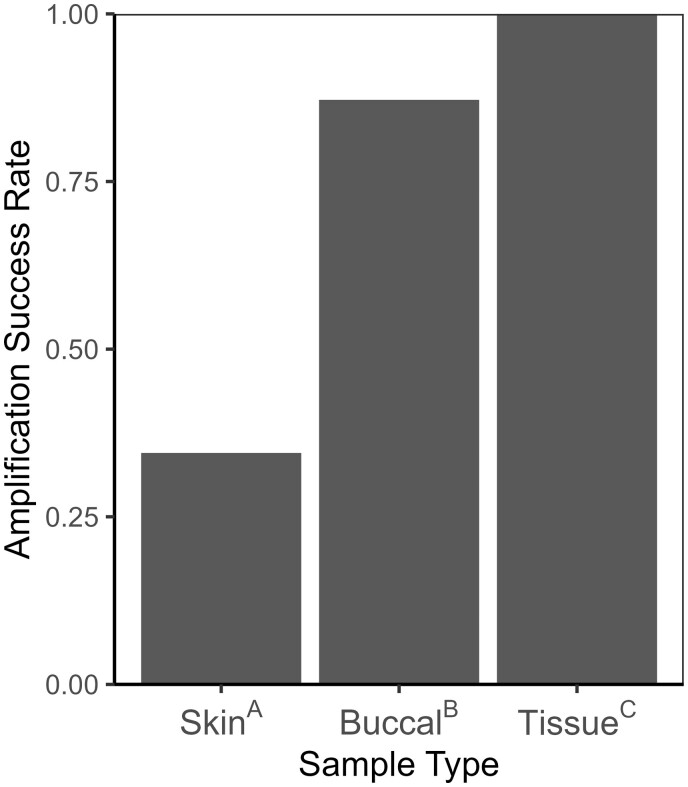
Amplification success rate (i.e. the proportion of PCR reactions that allowed for allele scoring) by sample type (skin and buccal swabs, tissue from toe clips), for Blanchard’s Cricket Frog (*A. blanchardi*) samples. Superscript letters after sample types indicate significant pairwise differences in amplification success rate between sample types, based on pairwise Fisher’s exact tests with Bonferroni correction (*P *≤* *.017).

All PCR reactions from toe tissues (*n* = 172) successfully amplified, which was a rate significantly greater than either swabbing method, as per pairwise Fisher’s exact tests (both *P *<* *.001; Fig. 3). Buccal swabs (*n* = 156) had the next highest amplification success rate (87.2%), which was significantly higher (*P *<* *.001) than the success rate (34.5%) of skin swabs (*n* = 168; Fig. 3). No amplification occurred for the Acr-28 marker for skin swab samples, but even discounting Acr-28, and considering only the amplification success of the other three markers (*n* = 126), the amplification success rate for skin swabs (46.0%) remained significantly lower than either buccal swabs or tissue samples (both *P *<* *.001). All other method–marker combinations, aside from Acr-28 for skin swabs, had some amount of successful amplification. Allele size estimates ranging from 304–424 base pairs (bp) were observed for marker Acr-14, 150–214 bp for Acr-3, 114–174 bp for Acr-2, and 383–431 bp for Acr-28 (see Table 1 for amplification success by method–marker combination).

**Table 1.** Breakdown of quality data by sampling method and microsatellite marker for Blanchard’s Cricket Frog (*A. blanchardi*) samples.

Sample type	Marker	*n*_AS_	AS	*n*_GE_	ADO	FA	GE
Skin swab	Acr-14	42	0.357	15	2	0	0.133
	Acr-3	42	0.571	24	3	6	0.375
	Acr-2	42	0.452	17	0	12	0.706
	Acr-28^a^	42	0	0	–	–	–
	Overall	168	0.345	56	5	18	0.411
Buccal swab	Acr-14	39	0.897	35	0	0	0
	Acr-3	39	0.923	36	0	4	0.111
	Acr-2	39	0.846	32	3	3	0.188
	Acr-28	39	0.821	32	1	1	0.063
	Overall	156	0.872	135	4	8	0.089
Toe clip	Acr-14	43	1	24	0	0	0
	Acr-3	43	1	24	0	0	0
	Acr-2	43	1	24	2	0	0.083
	Acr-28	43	1	24	0	0	0
	Overall	172	1	96	2	0	0.021

Marker—the microsatellite primers used (Beauclerc *et al*. [64]), *n*_AS—_sample size for amplification success rate analysis (i.e. the number of attempted initial PCR reactions); AS: amplification success rate; *n*_GE—_sample size for genotyping error rate analyses (i.e. the number of comparisons to corresponding tissue samples); ADO: allelic dropout count; FA: false allele count; GE: genotyping error rate.

a No skin swabs amplified for this marker.

**Figure 4.**
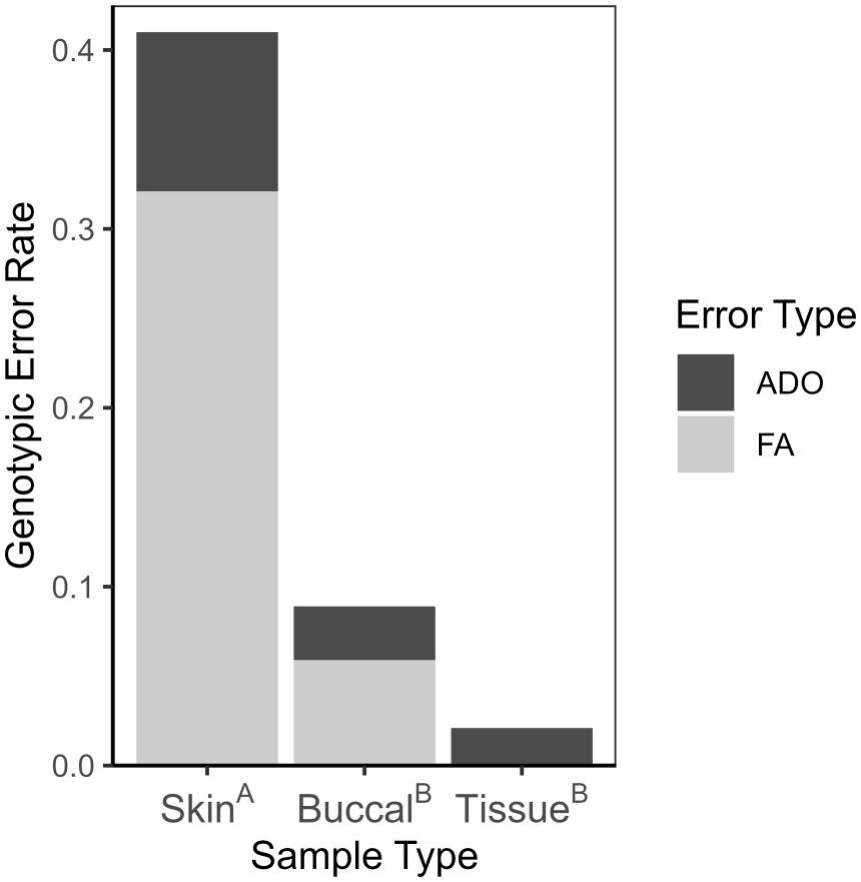
Genotyping error rate (i.e. the proportion of alleles that did not match corresponding tissue samples) by sample type (skin and buccal swabs, tissue from toe clips), for Blanchard’s Cricket Frog (*A. blanchardi*) samples. The proportion of each sample type’s error attributed to allelic dropout (ADO) and false alleles (FA) are symbolized. Superscript letters after sample types indicate significant pairwise differences in genotyping error rate between sample types, based on pairwise Fisher’s exact tests with Bonferroni correction (*P *≤* *.017).

Genotyping errors of buccal swabs occurred in 8.9% of comparisons (*n* = 135), which did not differ significantly (*P *=* *.047, Bonferroni-corrected α = 0.0167) with the 2.1% error rate of tissue comparisons (*n* = 96; Fig. 4). Skin swab comparisons (*n* = 56) had a genotyping error rate of 41.1%, which was significantly higher than the other two sample types (both *P *<* *.001; Fig. 4). False alleles were the most common kind of error for each sample method (see Table 1).

## Discussion

Less invasive sampling methods, and those which require less handling time are preferable for amphibian studies, as a high proportion of species in this vertebrate group are declining [66, 67]. However, we found common amphibian sampling methods cannot be expected to perform equally in all scenarios. Being the first direct comparison of the genotyping efficacy of buccal swabs, skin swabs, and toe clips, we generally found buccal swabs, but not skin swabs, to be a feasible alternative to tissue sampling for Blanchard’s Cricket Frogs. Buccal swabs were as effective as tissue samples by all measures, aside from buccal swabs having a significantly lower amplification success rate. Skin swabs significantly underperformed across all metrics, relative to samples from toe tissue and buccal swabs.

The poor performance of skin swabs suggests that this sampling method might be unsuitable for gathering reliable genetic data from amphibians. We found skin swabbing to yield roughly an eighth the amount of DNA as buccal swabbing or toe clipping, a disparity similar to that found by Prunier *et al*. [37] between skin and buccal swabs of European Tree Frogs (*Hyla arborea*), though other studies have found skin swabs to have comparable DNA yield with buccal swabs [32] and toe clips [33]. The varying body size of the focal species of these studies, and the fact that we obtained less DNA than Ringler [33], despite using a more vigorous swabbing protocol on a comparatively small species, suggests that surface area does not fully account for the mixed results for skin swab yield across the limited literature on this topic.

Skin swabs still, theoretically, provided enough templates for multiple PCR reactions using the kits implemented in this study. However, the NanoDrop does not differentiate target DNA from other DNA sources in samples, so skin swab DNA concentration measurements could have been comprised partly of DNA from microbiota and other nontarget DNA from the environment, inflating the yield measurement. Such nontarget DNA contamination has been hypothesized previously for skin swab techniques [33]. Additionally, we cannot confirm that our skin swab yields would be adequate for many next-generation sequencing approaches that require more templates than the microsatellite methods we applied [27, 50]. The use of commercial extraction kits, which we did not use, has been reported to result in slightly higher mean DNA yield from swab samples [32], but our use of a consistent extraction technique across sample types suggests that skin swabs can be expected to collect less DNA relative to buccal swabs or toe clips, at least in the case of *Acris*.

What is a larger concern than lower DNA yield for skin swabs is the significantly lower purity, amplification success rate, and genotyping accuracy we found for this method. Large disparities in amplification and genotyping success between skin swabbing and other sampling methods, like those we observed, have been reported [32, 33; but see 37]. Species and life stage are important factors when considering skin swabbing; Pichlmüller *et al*. [15] observed zero instances of false alleles or allelic dropout when skin swabbing Fire Salamanders (*Salamandra salamandra*) in their aquatic, larval form. While low yield and amplification rate problems might be adequately mitigated by repeated PCR reactions [68, 69], protocols employing replicated PCR reactions cause study costs to increase multiplicatively [12]. Furthermore, genotyping errors from conspecific contamination are possible with external swabbing [32] and could persist through repeated PCR reactions. The probability of conspecific contamination likely increases when conspecifics are densely grouped, like during breeding aggregations, when many amphibian studies occur. Unlike Ringler [33], who reported skin swabs to have purity comparable to tissues, we found skin swabs to be of significantly lower purity than buccal or toe samples, in terms of contaminants with absorbance of 280 nm (e.g. proteins), highlighting possible forms of contamination hindering this method. Post-extraction treatment of the samples with purification kits could resolve some of the issues associated with the lower purity of skin swabs we found, but their use may also result in inadequate amounts of DNA templates for PCR [33].

That skin swab template did not amplify for marker Acr-28 suggests that there could be marker-dependent implications for various sampling methods, given that other primers had comparable success rates across markers within a method type (Table 1). While Acr-28 had the largest alleles, and sequences of larger fragments are theoretically more likely to experience amplification-impeding degradation than shorter fragments [70, 71], a higher probability of degradation cannot explain the disproportionate failure of Acr-28, as the base pair range of Acr-14 is comparable to Acr-28. Ultimately, sampling method considerations might be further complicated by method–marker interactions.

We found false alleles to be more common than allelic dropout, and many of the false alleles we observed for skin swabs were allele peaks not matching corresponding toe clip heterozygote peaks (e.g. a skin swab and its corresponding tissue sample being heterozygotic for a given marker, with two completely different pairs of alleles), which could be evidence of conspecific contamination. Individuals coming into contact during the breeding season could be an obvious source of conspecific contamination, though Prunier *et al*. [37] skin swabbed anurans during breeding congregations but reported high-quality data from skin swabbing. There may be some life history or biological explanation related to Blanchard’s Cricket Frogs, not attributable to other amphibians, that might increase the risk of conspecific contamination with external swabbing. For example, it has been suggested the species can mate multiple times, with presumably multiple mates, in each breeding season [72], making them more susceptible to contamination than less-promiscuous or less-frequently breeding species. Physical contact during male-male territorial disputes, common for Blanchard’s Cricket Frogs [73, 74], could also introduce conspecific contamination. While the cause for the genotyping error is unknown, we do provide evidence refuting the previously proposed idea that more aquatic species are better suited for skin swabbing [33]. Blanchard’s Cricket Frogs did not generate effective skin swab samples, despite being the most aquatic North American treefrog species [53] and all individuals sampled for this work were caught within or within a few meters of waterbodies.

Contrary to skin swabs, buccal swabs appear to be an effective alternative to tissue sampling, with the caveat that buccal swabbing showed a significantly lower amplification success rate than toe clip samples. We found buccal swabs to exhibit low genotyping error, providing more evidence that buccal swabs generate genetic data of comparable quality to tissues [14, 34, 42]. We also found buccal swabs to have statistically equal DNA yields as toe clips, whereas Gallardo *et al*. [42] found a large disparity in yield between these sample types for a similarly small anuran; using flocked swabs for our buccal swabbing could have helped increase our yield with this method, like has been found for buccal swabbing reptiles [35]. However, we cannot be certain all the DNA yielded from buccal swabs was that of our target species because amphibian mouths host microbiomes [75, 76]. While amphibian oral microbiomes are far less studied than amphibian skin microbiomes [75], we cannot suggest bacteria are less abundant in Blanchard’s Cricket Frog mouths than on their skin. Still, our results suggest a lesser contamination risk exists for buccal swabbing than skin swabbing because buccal swabs outperformed skin swabs in all our quality assessments.

While buccal swabs had a significantly lower amplification success rate than tissue samples, the disparity between these rates (12.8%) was relatively small. We found the genotypic data quality from buccal swabs comparable to data from tissues, and the amplification failure rate of buccal swabs is also lower than acceptable proportions of missing data for many population and conservation genetics analyses [77, 78]. Researchers ought to consider using buccal swabbing in place of more invasive tissue sampling, especially when studying threatened and endangered species, though they should consider whether decreased amplification success would be an acceptable risk. If feasible, a pilot study to determine differences in amplification success could provide support for sampling method choice.

It has been hypothesized that buccal swabbing small species could be harmful [15, 33, 37], presumably based on the assumption that the bleeding reported by Pidancier *et al*. [14] while buccal swabbing larger species would be exacerbated for smaller species. It has been claimed that buccal swabbing small (<33 mm) amphibians is effective for producing genetic data [42], but the only direct reporting on the ethical concern of doing so, to our knowledge, is from Mudke *et al*. [46], who anecdotally reported individuals did not exhibit “discomfort.” While Mudke *et al*. [46] sampled an even smaller species than us, they immediately released sampled frogs. We can report that buccal swabbed Blanchard’s Cricket Frogs showed no signs of harm, as evidenced by observations that males and females continued normal movement and behavior within containers after sampling and upon release, and many males resumed calling soon after swabbing. Our ability to collect buccal swabs without a device to pry the mouth open [14, 37], likely minimized harm caused by sampling [27]. Researchers should confirm the need for such prying objects before using them. The absence of any bleeding or discomfort could also have been influenced by our using small-diameter (∼2.5 mm) swabs intended for human nasal sampling, which might be less likely to cause abrasions than the larger, synthetic- or cotton-tipped swabs commonly used [30, 37, 42].

While we assert buccal, but not skin swabs are likely an adequate alternative to tissue sampling, we also stress the importance of pilot studies to test sampling methods [14, 15, 33, 48]. Preliminary assessments can afford researchers insight into the effect of sampling methods on data quality, study feasibility and costs, and animal welfare. Furthermore, researchers ought to familiarize themselves with the handling of their study species and their sampling protocols; skin swabbing could be easier than alternative methods for inexperienced data collectors [36], but Pichlmüller *et al*. [15] found handling experience of the person collecting skin swabs to significantly impact DNA yield. Additionally, while a portion of studies provide brief statements on the impact of sampling, we encourage better reporting of the effects of nonlethal sampling on individuals, preferably by way of empirical assessments.

Questions remain regarding the efficacy of common amphibian sampling methods. It has been suggested that skin swabbing could be more efficacious after rinsing animals’ exteriors [32], which should be tested empirically, as this could be a simple step to make the arguably simpler and more ethical skin swab method more efficacious. The use of flocked swabs [33] or other kinds of swab material, such as FTA^®^ (Flinders Technology Associates) cards [50], could also improve skin swab performance. Further use of skin swabbing in herpetology could also benefit from empirically investigating how breeding aggregations might lead to conspecific contamination, and sampling a spectrum of life histories to identify how ecological and biological factors might affect method efficacy.

Assuming researchers value striving towards the “3 Rs” (replace, reduce, and refine) coined by Russel and Burch [1], less invasive sampling options should be favored, especially in the case of sensitive species, when resultant data quality is comparable to more invasive methods. Case-specific considerations should be made for all studies, but we generally assert buccal swabbing might often be advantageous over more invasive tissue collection and skin swabbing might often lead to low-quality data. Swabbing might also often be simpler in field settings [34] and easier for acquiring permits than tissue collection. Our results add to existing information on the effectiveness of common amphibian genetic sampling methods and highlight caveats that may be important to researchers based on their study systems, research questions, and study designs.

## Supplementary Material

bpae030_Supplementary_Data

## Data Availability

Data are available as supplementary files.

## References

[1] Russell WMS , Burch RL. The Principles of Humane Experimental Technique. London: Methuen and Company Limited, 1959.

[2] Beja-Pereira A , Oliveira R, Alves PC et al Advancing ecological understandings through technological transformations in noninvasive genetics. Mol Ecol Resour 2009;9:1279–301. 21564900 10.1111/j.1755-0998.2009.02699.x

[3] Carroll EL , Bruford MW, DeWoody JA et al Genetic and genomic monitoring with minimally invasive sampling methods. Evol Appl 2018;11:1094–119. 30026800 10.1111/eva.12600 PMC6050181

[4] Beng KC , Corlett RT. Applications of environmental DNA (eDNA) in ecology and conservation: opportunities, challenges and prospects. Biodivers Conserv 2020;29:2089–121.

[5] Heyer R , Donnelly MA, Foster M et al Measuring and Monitoring Biological Diversity: Standard Methods for Amphibians. Washington, DC: Smithsonian Institution Press, 2014.

[6] Schulte U , Gebhard F, Heinz L et al Buccal swabs as a reliable non-invasive tissue sampling method for DNA analysis in the lacertid lizard *Podarcis muralis*. North-West J Zool 2011;7:325–8.

[7] Billerman SM , Jesmer BR, Watts AG et al Testing theoretical metapopulation conditions with genotypic data from Boreal Chorus Frogs (*Pseudacris maculata*). Can J Zool 2019;97:1042–53.

[8] Mucci N , Mengoni C, Berti E et al Cloacal swab sampling is a reliable and harmless source of DNA for population and forensic genetics in tortoises. Conserv Genet Resour 2014;6:845–7.

[9] Cox K , Denoël M, Van Calster H et al Scale-dependent effects of terrestrial habitat on genetic variation in the great crested newt (*Triturus cristatus*). Landsc Ecol 2021;36:3029–48.

[10] Crawford AJ , Cruz C, Griffith E et al DNA barcoding applied to ex situ tropical amphibian conservation programme reveals cryptic diversity in captive populations. Mol Ecol Resour 2013;13:1005–18. 23280343 10.1111/1755-0998.12054

[11] Beninde J , Keltsch F, Veith M et al Connectivity of Alpine newt populations (*Ichthyosaura alpestris*) exacerbates the risk of *Batrachochytrium salamandrivorans* outbreaks in European fire salamanders (*Salamandra salamandra*). Conserv Genet 2021;22:653–9.

[12] Taberlet P , Waits LP, Luikart G. Noninvasive genetic sampling: look before you leap. Trends Ecol Evol 1999;14:323–7. 10407432 10.1016/s0169-5347(99)01637-7

[13] Pauli JN , Whiteman JP, Riley MD et al Defining noninvasive approaches for sampling of vertebrates. Conserv Biol 2010;24:349–52. 19624526 10.1111/j.1523-1739.2009.01298.x

[14] Pidancier N , Miquel C, Miaud C. Buccal swabs as a non-destructive tissue sampling method for DNA analysis in amphibians. Herpetol J 2003;13:175–8.

[15] Pichlmüller F , Straub C, Helfer V. Skin swabbing of amphibian larvae yields sufficient DNA for efficient sequencing and reliable microsatellite genotyping. Amphib Reptilia 2013;34:517–23.

[16] Zemanova MA. Towards more compassionate wildlife research through the 3Rs principles: moving from invasive to non‐invasive methods. Wildl Biol 2020;2020:1–17.

[17] Zemanova MA. Poor implementation of non-invasive sampling in wildlife genetics studies. Rethink Ecol 2019;4:119–32.

[18] Funk WC , Donnelly MA, Lips KR. Alternative views of amphibian toe-clipping. Nature 2005;433:193. 10.1038/433193c 15662387

[19] McCarthy M , Parris K. Identifying effects of toe clipping on Anuran return rates: the importance of statistical power. Amphib Reptilia 2001;22:275–89.

[20] Waddle JH , Rice KG, Mazzotti FJ et al Modeling the effect of toe clipping on treefrog survival: beyond the return rate. J Herpetol 2008;42:467–73.

[21] Schmidt K , Schwarzkopf L. Visible implant elastomer tagging and toe-clipping: effects of marking on locomotor performance of frogs and skinks. Herpetol J 2010;20:99–105.

[22] Fisher KJ , Guilfoyle KJ, Hatch KA. Stress induced by toe-clipping in cane toads (*Rhinella marina*). Copeia 2013;2013:539–42.

[23] Perry G , Wallace MC, Perry D et al Toe clipping of amphibians and reptiles: science, ethics, and the law. J Herpetol 2011;45:547–55.

[24] Zamora-Camacho FJ. Toe-clipping does not affect toad’s short-term locomotor performance. Ann Zool Fenn 2018;55:237–46.

[25] Zamora-Camacho FJ , Comas M, Pascual G et al The effect of toe-clipping on locomotor performance and return rates in a frog. S Am J Herpetol 2023;28:38–46.

[26] Lefort MC , Cruickshank RH, Descovich K et al Blood, sweat and tears: a review of non-invasive DNA sampling. Peer Community J 2022;2:e16.

[27] Ambu J , Dufresnes C. Buccal swabs for amphibian genomics. Amphib-Reptilia 2023;44:249–55.

[28] Davis A , Slusher LB, Reneski JT. A novel method for the extraction and amplification of DNA from amphibians. Herpetol Rev 2002;33:104–5.

[29] Goldberg CS , Kaplan ME, Schwalbe CR. From the frog's mouth: buccal swabs for collection of DNA from amphibians. Herpetol Rev 2003;34:220–1.

[30] Poschadel JR , Möller D. A versatile field method for tissue sampling on small reptiles and amphibians, applied to pond turtles, newts, frogs and toads. Conserv Genet 2004;5:865–7.

[31] Goodman RM , Miller DL, Ararso YT. Prevalence of ranavirus in Virginia turtles as detected by tail-clip sampling versus oral-cloacal swabbing. Northeast Nat 2013;20:325–32.

[32] Müller AS , Lenhardt PP, Theissinger K. Pros and cons of external swabbing of amphibians for genetic analyses. Eur J Wildl Res 2013;59:609–12.

[33] Ringler E. Testing skin swabbing for DNA sampling in dendrobatid frogs. Amphib Reptil 2018;39:245–51. 31327883 10.1163/15685381-17000206 PMC6640035

[34] de Almeida LM , Pavan KMC, de Carvalho MCCG et al Swab bucal em anuros: uma proposta de substituição ao uso de métodos invasivos para obtenção de DNA animal em pesquisa. Braz J Dev 2020;6:54064–72.

[35] Adair MG , Forgus JJ, Main DC et al The pros and cons of buccal swabbing and tail clipping for monitoring reptilian biodiversity. S Afr J Sci 2023;119:1–9.

[36] Robbemont J , van Veldhuijzen S, Allain SJ et al An extended mtDNA phylogeography for the alpine newt illuminates the provenance of introduced populations. Amphib-Reptilia 2023;44:347–61.

[37] Prunier J , Kaufmann B, Grolet O et al Skin swabbing as a new efficient DNA sampling technique in amphibians, and 14 new microsatellite markers in the alpine newt (*Ichthyosaura alpestris*). Mol Ecol Resour 2012;12:524–31. 22248363 10.1111/j.1755-0998.2012.03116.x

[38] Rothstein AP , Knapp RA, Bradburd GS et al Stepping into the past to conserve the future: archived skin swabs from extant and extirpated populations inform genetic management of an endangered amphibian. Mol Ecol 2020;29:2598–611. 32573039 10.1111/mec.15515

[39] Hinneberg H , Bamann T, Geue JC et al Truly invasive or simply non‐native? Insights from an artificial crested newt hybrid zone. Conserv Sci Pract 2022;4:e12752.

[40] Zakšek V , Konec M, Trontelj P. First microsatellite data on Proteus anguinus reveal weak genetic structure between the caves of Postojna and Planina. Aquat Conserv: Mar Freshw Ecosyst 2018;28:241–6.

[41] Reyne M , Helyar S, Aubry A et al Combining spawn egg counts, individual photo‐ID and genetic fingerprinting to estimate the population size and sex ratio of an endangered amphibian. Integr Zool 2021;16:240–54. 33137231 10.1111/1749-4877.12497

[42] Gallardo CE , Correa C, Morales P et al Validation of a cheap and simple nondestructive method for obtaining AFLPs and DNA sequences (mitochondrial and nuclear) in amphibians. Mol Ecol Resour 2012;12:1090–6. 22978706 10.1111/1755-0998.12013

[43] Nuñez JJ , Suárez-Villota EY, Quercia CA et al Phylogeographic analysis and species distribution modelling of the wood frog *Batrachyla leptopus* (Batrachylidae) reveal interglacial diversification in south western Patagonia. PeerJ 2020;8:e9980. 33083116 10.7717/peerj.9980 PMC7546244

[44] Othman SN , Choe M, Chuang MF et al Across the Gobi Desert: impact of landscape features on the biogeography and phylogeographically-structured release calls of the Mongolian Toad, *Strauchbufo raddei* in East Asia. Evol Ecol 2022;36:1007–43.

[45] Borzée A , Fong JJ, Nguyen HQ, Jang Y. Large-scale hybridisation as an extinction threat to the Suweon treefrog (Hylidae: *dryophytes suweonensis*). Animals 2020;10:764. 32349428 10.3390/ani10050764 PMC7278489

[46] Mudke MM , Aravind NA, Gururaja KV et al First report on the fossorial tadpole of *Micrixalus kottigeharensis* (Rao, 1937). Herpetol Notes 2020;13:645–8.

[47] Krug A , Auffarth J, Prohl H. Applying population genetics to define the units for conservation management in the European Tree Frog, *Hyla arborea*. Amphib Reptile Conserv 2022;16:69–87.

[48] Broquet T , Berset-Braendli L, Emaresi G et al Buccal swabs allow efficient and reliable microsatellite genotyping in amphibians. Conserv Genet 2007;8:509–11.

[49] Maddock ST , Lewis CJ, Wilkinson M et al Non-lethal DNA sampling for caecilian amphibians. Herpetol J 2014;24:255–60.

[50] Ward A , Hide G, Jehle R. Skin swabs with FTA^®^ cards as a dry storage source for amphibian DNA. Conserv Genet Resour 2019;11:309–11.

[51] Xu Y , Guan T, Liu J et al An efficient and safe method for the extraction of total DNA from shed frog skin. Conserv Genet Resour 2020;12:225–9.

[52] Folt BP , Davis JG. Blanchard’s cricket frog. In: Pfingsten RA, Davis JG, Matson TO et al (eds), Amphibians of Ohio. Columbus, OH: Ohio Biological Survey, 2013, 495–509.

[53] Ralin DB , Rogers JS. Aspects of tolerance to desiccation in *Acris crepitans* and *Pseudacris streckeri*. Copeia 1972;1972:519–25.

[54] Rainey T. Persistence and population genetics of a threatened frog in a fragmented landscape. Master’s Thesis, Central Michigan University, 2023.

[55] Zanatta DT , Wilson CC. Testing congruency of geographic and genetic population structure for a freshwater mussel (Bivalvia: unionoida) and its host fish. Biol J Linn Soc 2011;102:669–85.

[56] Matlock B. Assessment of Nucleic Acid Purity. Thermo Fisher Scientific. Technical Note 52646. 2015. https://assets.thermofisher.com/TFS-Assets/CAD/Product-Bulletins/TN52646-E-0215M-NucleicAcid.pdf (5 February 2024, date last accessed).

[57] Yu S , Wang Y, Li X et al The factors affecting the reproducibility of micro-volume DNA mass quantification in Nanodrop 2000 spectrophotometer. Optik 2017;145:555–60.

[58] García-Alegría AM , Anduro-Corona I, Pérez-Martínez CJ et al Quantification of DNA through the NanoDrop spectrophotometer: methodological validation using standard reference material and Sprague Dawley rat and human DNA. Int J Anal Chem 2020;2020:8896738. 33312204 10.1155/2020/8896738 PMC7719535

[59] Wickham H , François R, Henry L et al *dplyr: A Grammar of Data Manipulation*. R package version 1.1.2. 2023.

[60] Ogle DH , Doll JC, Wheeler AP et al *FSA: Simple Fisheries Stock Assessment Methods*. R package version 0.9.5. 2023.

[61] R Core Team. *R: A Language and Environment for Statistical Computing.* Vienna, Austria: R Core Team, 2020.

[62] Wickham H. *ggplot2: Elegant Graphics for Data Analysis.* R package version 3.5.0. 2016*.*

[63] RStudio Team. *RStudio: Integrated Development Environment for R.* Boston, MA: RStudio, PBC. 2021.

[64] Beauclerc KB , Johnson B, White BN. Characterization, multiplex conditions, and cross‐species utility of tetranucleotide microsatellite loci for Blanchard’s Cricket Frog (*Acris crepitans blanchardi*). Mol Ecol Notes 2007;7:1338–41.

[65] Nakazawa M. *fmsb: Functions for Medical Statistics Book with some Demographic Data.* R package version 0.7.3. 2022.

[66] Stuart SN , Chanson JS, Cox NA et al Status and trends of amphibian declines and extinctions worldwide. Science 2004;306:1783–6. 15486254 10.1126/science.1103538

[67] Campbell Grant EH , Miller DA, Muths E. A synthesis of evidence of drivers of amphibian declines. Herpetologica 2020;76:101–7.

[68] Navidi W , Arnheim N, Waterman M. A multiple-tubes approach for accurate genotyping of very small DNA samples by using PCR: statistical considerations. Am J Hum Genet 1992;50:347–59. 1734715 PMC1682471

[69] Taberlet P , Griffin S, Goossens B et al Reliable genotyping of samples with very low DNA quantities using PCR. Nucleic Acids Res 1996;24:3189–94. 8774899 10.1093/nar/24.16.3189 PMC146079

[70] Takahashi M , Kato Y, Mukoyama H et al Evaluation of five polymorphic microsatellite markers for typing DNA from decomposed human tissues: correlation between the size of the alleles and that of the template DNA. Forensic Sci Int 1997;90:1–9. 9438360 10.1016/s0379-0738(97)00129-1

[71] Alaeddini R , Walsh SJ, Abbas A. Forensic implications of genetic analyses from degraded DNA—a review. Forensic Sci Int Genet 2010;4:148–57. 20215026 10.1016/j.fsigen.2009.09.007

[72] Witte K , Chen KC, Wilczynski W et al Influence of amplexus on phonotaxis in the cricket frog *Acris crepitans blanchardi*. Copeia 2000;2000:257–61.

[73] Wagner WE. Fighting, assessment, and frequency alteration in Blanchard’s Cricket Frog. Behav Ecol Sociobiol 1989;25:429–36.

[74] Horne EA , Foulks S, Bello NM. Visual display in Blanchard’s Cricket Frogs (*Acris blanchardi*). Southwest Nat 2014;59:409–13.

[75] Mann MB , Prichula J, de Castro MS et al The oral bacterial community *Melanophryniscus admirabilis* (admirable red-belly toads): implications for conservation. Microorganisms 2021;9:220. 33499099 10.3390/microorganisms9020220 PMC7912307

[76] Zhu W , Zhao C, Feng J et al Effects of habitat river microbiome on the symbiotic microbiota and multi-organ gene expression of captive-bred Chinese giant salamander. Front Microbiol 2022;13:884880. 35770173 10.3389/fmicb.2022.884880 PMC9234736

[77] Smith O , Wang J. When can noninvasive samples provide sufficient information in conservation genetics studies? Mol Ecol Resour 2014;14:1011–23. 24620908 10.1111/1755-0998.12250

[78] Hodel RG , Chen S, Payton AC et al Adding loci improves phylogeographic resolution in red mangroves despite increased missing data: comparing microsatellites and RAD-Seq and investigating loci filtering. Sci Rep 2017;7:17598. 29242627 10.1038/s41598-017-16810-7 PMC5730610

